# Unlocking the Molecular Secrets of Antifolate Drug Resistance: A Multi-Omics Investigation of the NCI-60 Cell Line Panel

**DOI:** 10.3390/biomedicines11092532

**Published:** 2023-09-14

**Authors:** Blake R. Rushing

**Affiliations:** 1 Nutrition Research Institute, University of North Carolina at Chapel Hill, Kannapolis, NC 28081, USA; blake_rushing@unc.edu; 2 Department of Nutrition, University of North Carolina at Chapel Hill, Chapel Hill, NC 27599, USA; 3 Department of Pathology and Laboratory Medicine, University of North Carolina at Chapel Hill, Chapel Hill, NC 27599, USA; 4 Lineberger Comprehensive Cancer Center, University of North Carolina at Chapel Hill, Chapel Hill, NC 27599, USA

**Keywords:** antifolates, drug resistance, multi-omics, metabolism, metabolomics, transcriptomics, proteomics, DNA methylation, copy number variants, cancer

## Abstract

Drug resistance continues to be a significant problem in cancer therapy, leading to relapse and associated mortality. Although substantial progress has been made in understanding drug resistance, significant knowledge gaps remain concerning the molecular underpinnings that drive drug resistance and which processes are unique to certain drug classes. The NCI-60 cell line panel program has evaluated the activity of numerous anticancer agents against many common cancer cell line models and represents a highly valuable resource to study intrinsic drug resistance. Furthermore, great efforts have been undertaken to collect high-quality omics datasets to characterize these cell lines. The current study takes these two sources of data—drug response and omics profiles—and uses a multi-omics investigation to uncover molecular networks that differentiate cancer cells that are sensitive or resistant to antifolates, which is a commonly used class of anticancer drugs. Results from a combination of univariate and multivariate analyses showed numerous metabolic processes that differentiate sensitive and resistant cells, including differences in glycolysis and gluconeogenesis, arginine and proline metabolism, beta-alanine metabolism, purine metabolism, and pyrimidine metabolism. Further analysis using multivariate and integrated pathway analysis indicated purine metabolism as the major metabolic process separating cancer cells sensitive or resistant to antifolates. Additional pathways differentiating sensitive and resistant cells included autophagy-related processes (e.g., phagosome, lysosome, autophagy, mitophagy) and adhesion/cytoskeleton-related pathways (e.g., focal adhesion, regulation of actin cytoskeleton, tight junction). Volcano plot analysis and the receiver operating characteristic (ROC) curves of top selected variables differentiating Q1 and Q4 revealed the importance of genes involved in the regulation of the cytoskeleton and extracellular matrix (ECM). These results provide novel insights toward mechanisms of intrinsic antifolate resistance as it relates to interactions between nucleotide metabolism, autophagy, and the cytoskeleton. These processes should be evaluated in future studies to potentially derive novel therapeutic strategies and personalized treatment approaches to improve antifolate response.

## 1. Introduction

Cancer drug resistance represents a formidable obstacle that severely impacts patient outcomes. The emergence of intrinsic resistance, preexisting prior to treatment initiation, as well as acquired resistance, acquired during or after therapy, significantly diminishes the efficacy of not only conventional chemotherapeutic agents but also novel tumor-targeted and immunotargeted therapies [1]. The current state of drug resistance in cancer treatment bears a substantial burden, as it is closely associated with disease relapse and heightened morbidity and mortality rates [2]. Despite remarkable advancements in cancer therapeutics, a considerable number of patients experience therapeutic failure due to the acquisition of drug resistance. Consequently, an intricate understanding of the underlying mechanisms facilitating cancer cell evasion and survival in the face of therapeutic interventions is imperative. The complex interplay between patient-specific factors, tumor heterogeneity, and the intricate tumor microenvironment contributes significantly to the multifaceted development and persistence of drug resistance. This interplay engenders a remarkable degree of inter- and intra-patient variability in treatment response, necessitating comprehensive investigations into the mechanisms underlying drug resistance [3,4,5,6]. To improve cancer outcomes, a comprehensive elucidation of the molecular and cellular determinants of drug resistance is warranted. By deciphering the intricate molecular signatures and pathways contributing to resistance phenotypes, novel therapeutic targets can be identified, and therapeutic strategies can be designed to circumvent or suppress resistance mechanisms.

One of the most commonly used chemotherapeutic drug classes is antifolates. Antifolate drugs play a crucial role in cancer therapy by inhibiting folate metabolism, which is essential for DNA synthesis and cell proliferation. These drugs target enzymes involved in the folate pathway, such as dihydrofolate reductase (DHFR) and thymidylate synthase (TS), thereby disrupting nucleotide production and impairing cancer cell growth [7]. However, resistance to antifolate drugs poses a significant challenge in cancer treatment. Multiple mechanisms contribute to resistance, including alterations in drug transporters, changes in target enzymes, and alterations in folate metabolism. One common mechanism is the overexpression or amplification of DHFR or TS genes, leading to increased enzymatic activity and reduced drug sensitivity. Additionally, mutations in the drug target sites can impair drug binding and decrease drug effectiveness. Other resistance mechanisms involve changes in the cellular folate metabolism machinery, such as increased folate uptake, enhanced intracellular folate pools, or the activation of alternative salvage pathways [8]. These alterations can bypass the antifolate drug action and sustain cancer cell survival and growth. Moreover, the tumor microenvironment and the heterogeneity of cancer cells contribute to the development of antifolate resistance. Factors such as hypoxia, nutrient availability, and interactions with stromal cells can impact drug response and promote resistance [8,9]. Despite advancements in understanding antifolate resistance, several gaps remain in elucidating the comprehensive molecular mechanisms and how molecular features of cancer cells can be used to predict drug response for optimal treatment regimens.

The advent of high-throughput omics technologies, encompassing epigenomics, genomics, proteomics, and metabolomics, and others, has provided unprecedented opportunities to unravel the complex molecular landscape associated with drug resistance in cancer. Utilizing these multi-omics approaches allows for the identification of key molecular signatures and networks implicated in drug resistance, thereby enabling the development of personalized treatment modalities tailored to individual patients [10]. Additionally, the integration of multi-omics data with advanced computational modeling holds promise for the identification of synergistic therapeutic combinations targeting multiple resistance mechanisms simultaneously [11]. The National Cancer Institute’s (NCI)-60 cancer cell line program contains the anticancer activity of thousands of small molecules [12]. Additionally, baseline molecular profiles have been previously collected for the cancer cell lines in this panel, and they are publicly available. The current investigation uses these existing omics datasets of NCI-60 cell lines along with response data of these cell lines to antifolate compounds to determine molecular networks that differentiate sensitive and resistant cells to antifolates. This information can be used to better understand the cellular and molecular determinants of response to this drug class, potentially identifying markers that can be used to predict drug response or targets that can be pursued to increase drug efficacy.

## 2. Materials and Methods

### 2.1. Procurement of Publicly Available Omics Datasets of the NCI-60 Cell Line Panel

All baseline omics datasets of NCI-60 cell lines were acquired from publicly available datasets. Transcriptomics data were obtained from https://www.ncbi.nlm.nih.gov/sites/GDSbrowser?acc=GDS4296, and replicate measurements were averaged [13,14] (accessed on 24 May 2023). Proteomics data were obtained from the Proteomics IDEntifications Database (PRIDE, https://www.ebi.ac.uk/pride/archive/projects/PXD013615) (accessed on 25 May 2023) [15]. Metabolomics data representing an average of triplicate experiments were obtained from the NCI’s Development Therapeutics Program (DTP) website (https://dtp.cancer.gov) (accessed on 24 May 2023). Lastly, copy number variant (CNV) data and DNA methylation data (averaged by gene) were downloaded from CellMiner (accessed on 21 July 2023) [16,17]. Two cell lines (MDA-N and SK-MEL-2) were absent in the metabolomics dataset and were therefore removed from the analysis.

### 2.2. Assessment of Antifolate Drug Response and Calculation of Overall Antifolate Sensitivity for the NCI-60 Cell Line Panel

Drug response data for all compounds screened in the NCI-60 cell line program were downloaded from CellMiner in the form of z-scores which are autoscaled GI_50_ values for each compound for each cell line [17]. Mechanism of action information for each screened compound in the NCI-60 cell line dataset was also downloaded from CellMiner to determine compounds with antifolate mechanisms. Compounds that did not have experimental data that passed NCI’s quality control parameters were not included in the analysis. Z-scores of each antifolate compound across cell lines were transformed into rank orders (without breaking ties). MetaboAnalyst 5.0 was used to generate a heatmap of z-score rank orders of all antifolate compounds across all cell lines to determine patterns of antifolate sensitivities in the cell line panel [18]. For each cell line, the median rank order across all antifolate compounds was calculated (without breaking ties) to derive an overall antifolate rank order, which was used to divide cell lines into quartiles based on antifolate sensitivity.

### 2.3. Univariate Analysis and Network Analysis of Features Differentiating Cell Lines Sensitive or Resistant to Antifolates

Fold changes and *p*-values were calculated for metabolites, proteins, and transcripts between cell lines in the top quartile (Q4, most resistant) and the bottom quartile (Q1, most sensitive). Fold changes were calculated using average values in Q4 and average values in Q1. *p*-values were calculated using Students’ *t*-test (two-tailed). Metabolites (as KEGG IDs), proteins (as Uniprot IDs), and transcripts (as official gene symbols) with a *p* < 0.1 were input into OmicsNet for integrated network analysis [19]. KEGG was used for database selection to map metabolite–protein interactions. Using the entire resulting network that was built, pathway analysis was performed using the KEGG (gene/protein) database [20].

### 2.4. Multi-Omic Multivariate Analysis and Joint Pathway Analysis of Sensitive versus Resistant Cell Lines to Antifolates

Relative abundance values for metabolomics, transcriptomics, proteomics, CNVs, and DNA methylation datasets were imported into Omicsanalyst for integrated multivariate analysis [21]. Quartile of antifolate sensitivity information for each cell line was also entered as metadata information. Autoscaling was performed on all datasets to unify data distribution patterns. Principal Component Analysis (PCA) was performed on each individual omics dataset in Omicsanalyst. Data Integration Analysis for Biomarker discovery using Latent variable approaches for Omics studies (DIABLO) was used for integrated analysis. DIABLO, a supervised multivariate method, uses a multi-block partial least squares-discriminant analysis (PLS-DA) approach to discriminate samples based on class information using high-dimensional data and identify variables which contribute to group separations [22]. DIABLO was used to identify variables that differentiated Q1 and Q4 samples based on loading scores. Joint pathway analysis was performed using MetaboAnalyst 5.0 on metabolites and transcripts as well as metabolites and proteins identified as differentiators by the DIABLO model. MetaboAnalyst 5.0′s “Statistical Analysis [one factor]” module was also used to generate volcano plots of DIABLO-selected variables between Q1 and Q4. The “Biomarker Analysis” module of MetaboAnalyst 5.0 was used to generate receiver operating characteristic (ROC) curves of DIABLO-selected variables to evaluate their ability to predict Q1 or Q4 status.

### 2.5. Pathview Analysis for Integrative Pathway Visualization

In order to visualize pathway perturbations using multi-omic data, the Pathview software https://pathview.uncc.edu/overview was utilized, incorporating data from metabolomics, proteomics, and transcriptomics datasets [23,24]. The average values in Q1 and Q4 cells were input into the software to be used to determine upregulated or downregulated variables. Only variables with a *p*-value less than 0.1 were included in the analysis. For the species database, hsa-Homo sapiens was selected. Positive fold changes were indicative of an increase in Q4 of the total rank order of z-scores, representing variables that increased in cells more resistant to antifolate treatment.

## 3. Results

**Figure 1:**
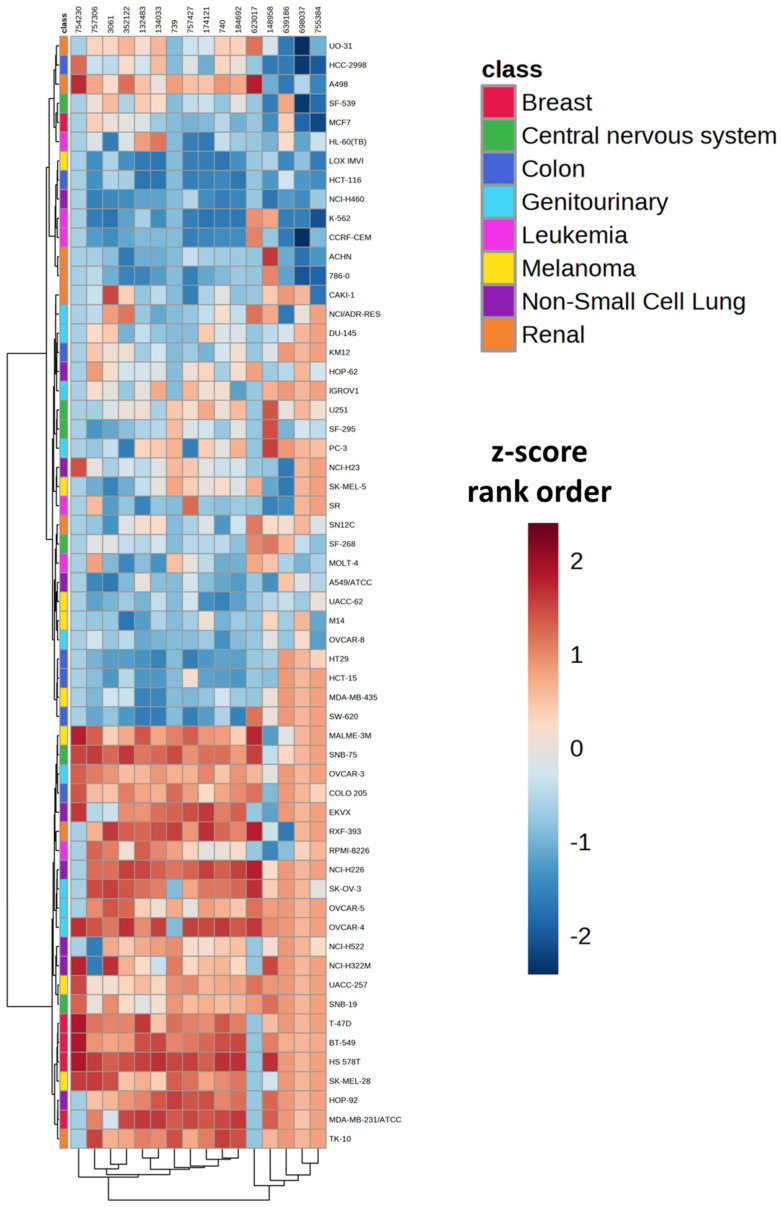
Heatmap representation of the rank order of z-scores for 16 antifolate agents across 58 cell lines within the NCI-60 cell line panel. Orange colors represent higher rank order values (more resistant), whereas blue colors represent lower rank order values (more sensitive). The distance measures were computed using the Euclidean method, and clustering was performed utilizing the Ward method in MetaboAnalyst 5.0. The compounds are identified by NSC identifiers, which are unique accession numbers assigned to each compound by the NCI.

Included in the NCI-60 cell line GI_50_ response data were sixteen entries for compounds that were defined by CellMiner as having an antifolate mechanism of action. These were broadly defined as antifols as well as inhibitors of specific enzymes including dihydrofolate reductase (DHFR), thymidylate synthetase (TYMS) inhibitors, and phosphoribosylglycinamide formyltransferase (GART) (Supplemental Table S1). The distribution of GI_50_ z-score rank orders across cell lines was visualized by heatmap in MetaboAnalyst 5.0 (Figure 1). Unsupervised clustering of cell lines in the heatmap revealed that the distribution of antifolate response was not driven by tissue type.

A comprehensive ranking of z-scores was computed by averaging the z-score values for all 16 antifolate agents across each cell line. This overall ranking was then used to categorize the 58 cell lines into quartiles. The fourth quartile (Q4) represented the cell lines with the highest z-score rank, indicating greater resistance to antifolate agents, while the first quartile (Q1) represented the cell lines with the lowest z-score rank, indicating higher sensitivity to antifolate agents (Table 1). Using these definitions, fold changes and *p*-values for all metabolites, transcripts, proteins, CNVs, and DNA methylation values were calculated between Q1 and Q4 (Supplemental Table S2).

**Figure 2:**
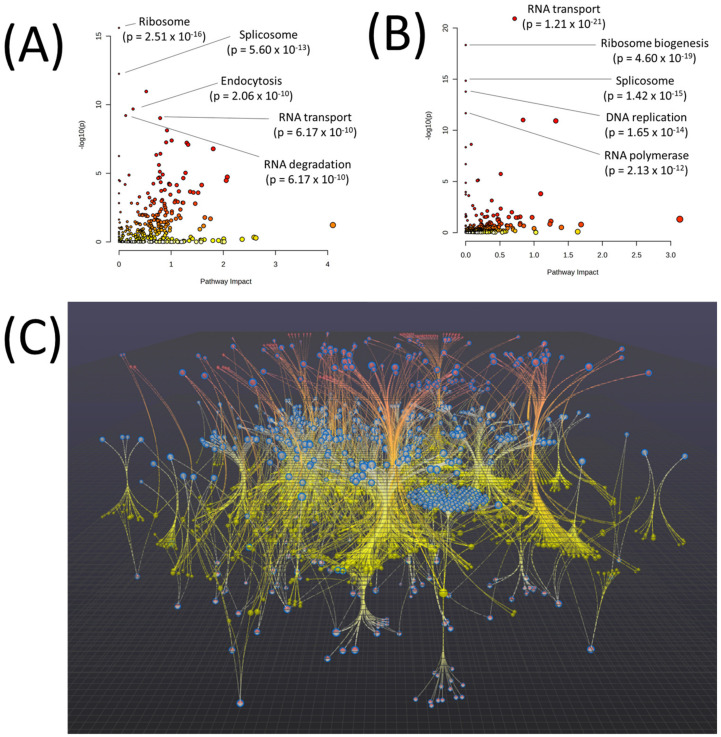
(**A**) Joint pathway analysis of metabolites and transcripts with *p* < 0.1 between Q1 and Q4. (**B**) Joint pathway analysis of metabolites and proteins with *p* < 0.1 between Q1 and Q4. Red points on the graph indicate a more significant pathway *p*-value. (**C**) Network analysis integrating metabolites, transcripts, and proteins, using variables with a significance level of *p* < 0.1 between Q1 and Q4 of the total rank order of z-scores. The network was constructed using Omicsnet, establishing metabolite-protein interactions through mapping to the KEGG database. Gray nodes in the network represent transcripts, red nodes represent proteins, and yellow nodes represent metabolites. Additionally, red and gray nodes indicate molecules present in both the transcriptomics and proteomics datasets. Seed nodes are highlighted with blue orders.

Joint pathway analysis was performed using metabolites, transcripts, and proteins with *p* < 0.1 between Q1 and Q4 to determine the overall differentiating cellular processes between the two groups. Joint pathway analysis with metabolites and transcripts revealed ribosome (*p* = 2.51 × 10^−16^), splicosome (*p* = 5.60 × 10^−13^), endocytosis (*p* = 2.06 × 10^−10^), RNA transport (*p* = 6.17 × 10^−10^), and RNA degradation (*p* = 6.17 × 10^−10^) as among the most significant pathways differentiating Q1 and Q4 (Figure 2A). Joint pathway analysis using metabolites and proteins revealed RNA transport (*p* = 1.21 × 10^−21^), ribosome biogenesis (*p* = 4.60 × 10^−19^), splicosome (*p* = 1.42 × 10^−15^), DNA replication (*p* = 1.65 × 10^−14^), and RNA polymerase (*p* = 2.13 × 10^−12^) as among the most significant pathways differentiating Q1 and Q4 (Figure 2B). To identify a focused set of molecules and pathways that distinguished between Q1 and Q4, Omicsnet—a powerful tool for integrated pathway network analysis using the KEGG database—was employed using metabolites, transcripts, and proteins with *p* < 0.1 between Q1 and Q4 (see Figure 2). This approach leverages established biological relationships among proteins, transcripts, and metabolites, constructing a core group of molecules based on the input lists. By doing so, the pathway analyses prioritize molecules that exhibit meaningful connections with each other, effectively excluding outlier molecules lacking strong associations with the overall set, while also considering “guilt by association” molecules that are known to interact with the input list [19]. This method ensures a more refined and relevant analysis of pathways related to the observed differences between Q1 and Q4. Results from the network analysis identified 86 pathways with an FDR-corrected *p*-value < 0.05 (full lists of all pathways from Figure 2 can be found in Supplemental Table S3). The top fifteen pathways from this analysis contained several resistance pathways as well as multiple metabolic processes related to nucleotides (purine and pyrimidine metabolism), carbohydrates (glycolysis/gluconeogenesis, pentose phosphate pathway), and select amino acids/peptides (arginine, proline, lysine, histidine, and glutathione metabolism) (Table 2).

**Figure 3:**
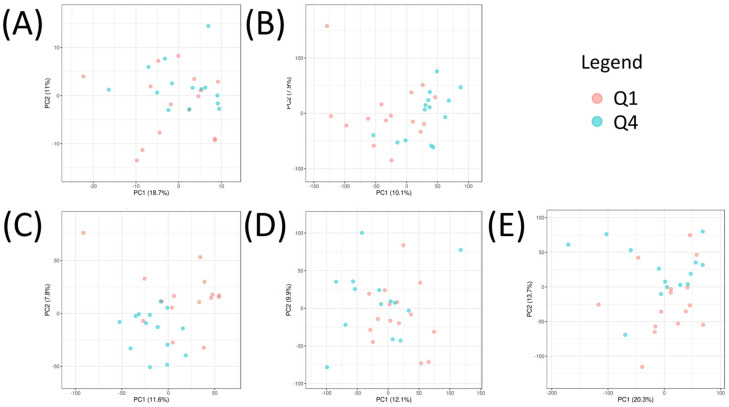
PCA of (**A**) metabolomics, (**B**) transcriptomics, (**C**) proteomics, (**D**) CNVs, and (**E**) DNA methylation values of Q1 (red) and Q4 (blue) samples. All datasets were autoscaled.

**Figure 4:**
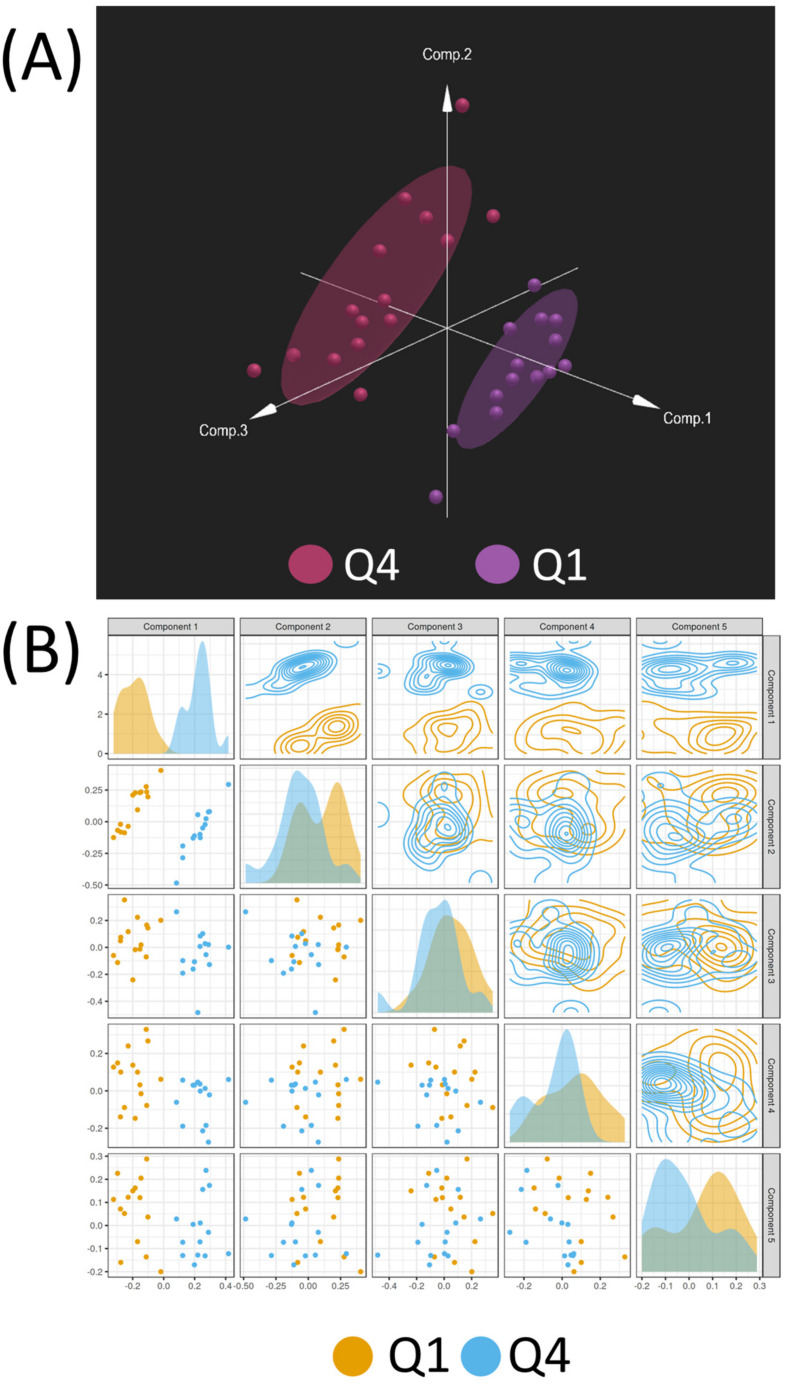
(**A**) DIABLO plot illustrating the clear demarcation between Q1 (purple) and Q4 (red) samples based on the integration of metabolomics, transcriptomics, proteomics, CNVs, and DNA methylation data. The plot demonstrates the effective separation of the two groups, reflecting distinct molecular profiles associated with Q1 and Q4 samples. (**B**) Summary of the first 5 components of the DIABLO model, showcasing the individual contribution of each component to the separation of Q1 and Q4 samples. All data were autoscaled.

To unravel the intricate relationship between the omics datasets and pinpoint the most influential combination of predictors distinguishing Q1 and Q4, a multivariate analysis was conducted using Omicsanalyst. Initially, each omics dataset (metabolomics, transcriptomics, proteomics, CNVs, and DNA methylation) was analyzed separately using PCA. This analysis revealed the separation of Q1 and Q4 in each dataset in an unsupervised manner (Figure 3A–E). Results showed the strongest visual separation of Q1 and Q4 in the proteomics, transcriptomics, and DNA methylation datasets, whereas the separation in the metabolomics and CNV datasets was more modest. DIABLO was then used to perform an integrated, supervised analysis between Q1 and Q4. The DIABLO analysis revealed a clear separation between Q1 and Q4 with the first component of the model exhibiting the most robust separation of the two groups (Figure 4A,B). For each variable (metabolite, transcript, protein, CNV, methylated gene), loading scores were obtained using this model. A cutoff of >|0.6| for the loadings value in the first component was applied to identify significant variables. As a result, 18 metabolites, 567 transcripts, 69 proteins, 118 CNVs, and 43 methylated genes were identified as key factors contributing to the differentiation between the two groups (Supplementary Table S4).

**Figure 5:**
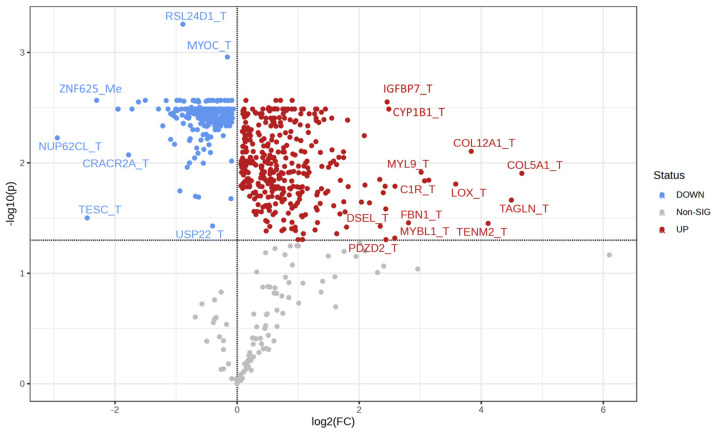
Volcano plot of variables with loadings values > |0.6| in PC1 of the DIABLO model. Colored dots represent variables with an FDR-adjusted *p*-value < 0.05 between Q1 and Q4 samples. Red dots represent upregulated variables, and blue dots represent downregulated variables in Q4 as compared to Q1. Variables ending in “_T” indicate those that are derived from the transcriptomics dataset, and variables ending in “_Me” indicate those that are derived from the DNA methylation dataset.

**Figure 6:**
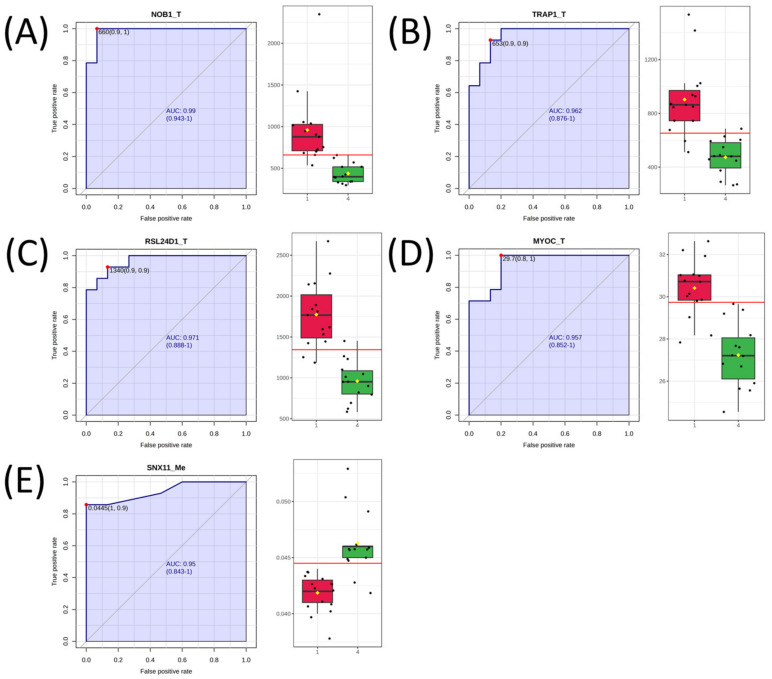
ROC curves of the top 5 DIABLO-selected variables that predict antifolate quartile status. (**A**) ROC curve of NOB1. (**B**) ROC curve of TRAP1. (**C**) ROC curve of RSL24D1. (**D**) ROC curve of MYOC. (**E**) ROC curve of SNX11. Variables ending in “_T” indicate those that are derived from the transcriptomics dataset, and variables ending in “_Me” indicate those that are derived from the DNA methylation dataset. Boxplots to the right of each ROC curve show the distribution of each variable in Q1 (red) and Q4 (green) samples.

Additional analyses were performed on DIABLO-selected variables. Firstly, volcano plot analysis was performed to identify molecules most significantly increased or decreased in Q4 cells. Results showed that transcripts overall had the largest fold changes between Q1 and Q4, with many genes related to adhesion/cytoskeletal structure (MYOC, COL12A1, COL5A1, MYL9, LOX, TAGLN, FBN1, TENM2, DSEL) and other notable differences in nuclear functions (NUP62CL, USP22, MYBL1) in calcium regulation (CRACR2A, TESC) (Figure 5). Receiver operating characteristic (ROC) curves were generated for each DIABLO-selected variable to determine their ability to predict Q4 samples. Twenty-five variables had an area under the curve (AUC) > 90% (Table 3) with transcript levels of NOB1, TRAP1, RSL24D1, and MYOC as well as the methylation status of SNX11 as the top 5 variables by AUC (Figure 6).

**Figure 7:**
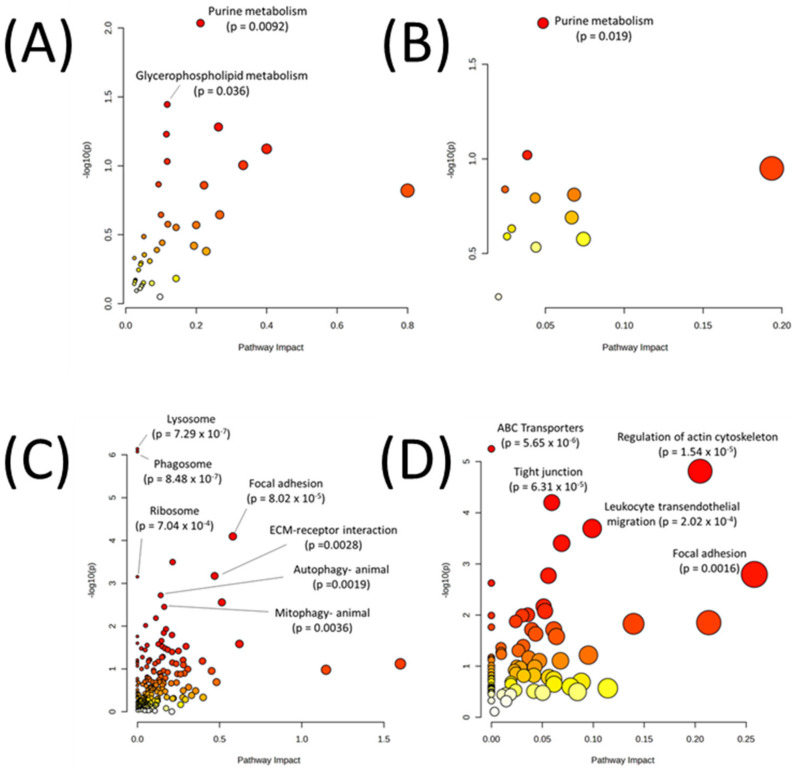
Joint pathway analysis of DIABLO-selected (**A**) metabolites and transcripts mapped to only metabolic pathways, (**B**) metabolites and proteins mapped to only metabolic pathways, (**C**) metabolites and transcripts mapped to all pathways, and (**D**) metabolites and proteins mapped to all pathways. Red points on the graph indicate a more significant pathway *p*-value.

**Figure 8:**
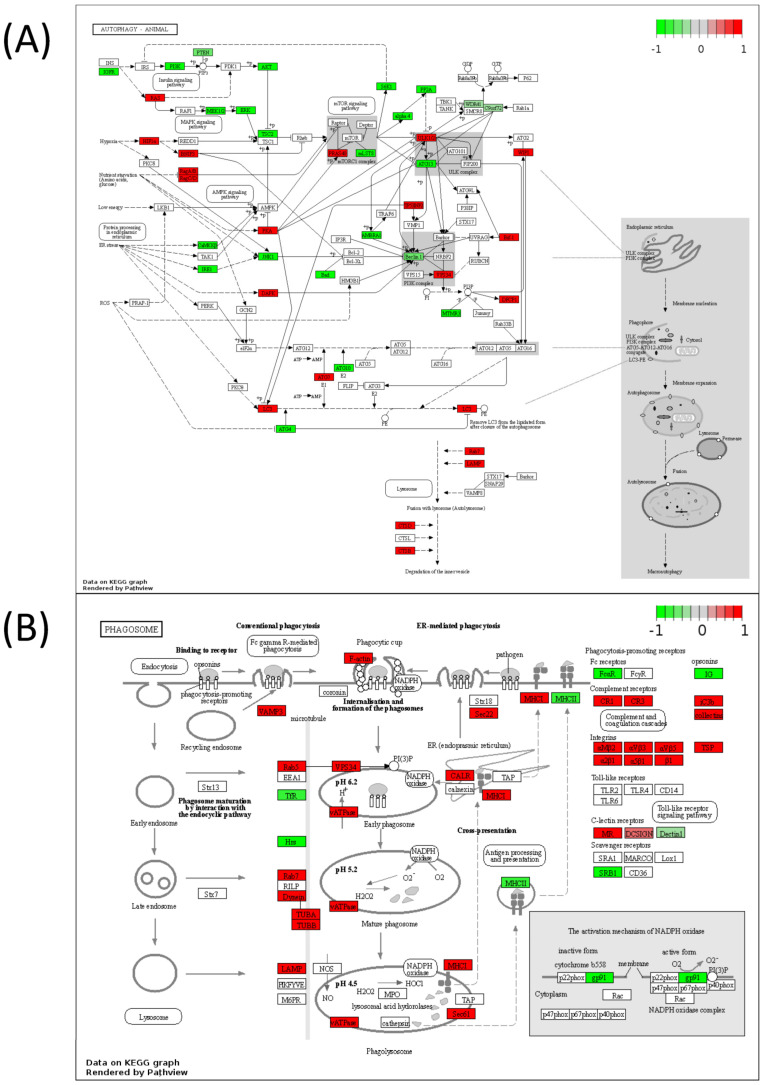
Pathview maps of (**A**) autophagy—animal and (**B**) phagosome KEGG pathways using variables with *p* < 0.1 between Q1 and Q4. Genes in red are increased in Q4, whereas genes in green are decreased in Q4.

**Figure 9:**
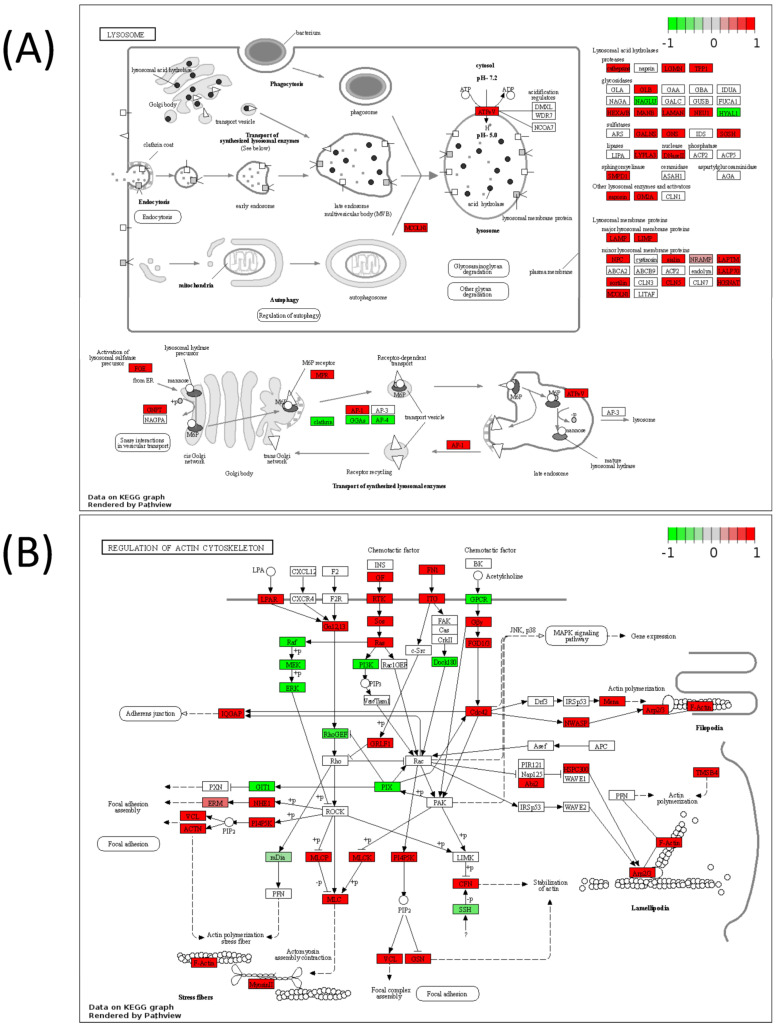
Pathview maps of (**A**) lysosome, and (**B**) regulation of actin cytoskeleton KEGG pathways using variables with *p* < 0.1 between Q1 and Q4. Genes in red are increased in Q4, whereas genes in green are decreased in Q4.

Lastly, pathway analyses were performed by mapping DIABLO-selected variables to metabolic pathways only or to all pathways to better understand the cellular function of these molecules. Joint pathway analysis utilizing the selected metabolites and transcripts from DIABLO (Figure 7A, Table 4) or the selected metabolites and proteins (Figure 7B, Table 5) demonstrated that purine metabolism emerged as the top significant metabolic pathway in both analyses. When mapping to all KEGG pathways, metabolites and transcripts (Figure 7C, Table 4) and metabolites and proteins (Figure 7D, Table 5) showed a significant enrichment in autophagy-related pathways (autophagy—animal, mitophagy—animal, lysosome, phagosome) and cell-adhesion/structural-related pathways (focal adhesion, ECM–receptor interaction, regulation of actin cytoskeleton, tight junction, focal adhesion, leukocyte transendothelial migration). Full pathways result tables for Figure 7 can be found in Supplementary Table S5. Many of these pathways were visualized using Pathview using metabolites, transcripts, and proteins with *p* < 0.1 between Q1 and Q4 to understand the overall increases or decreases in the activities of these pathways in Q4 samples (Figure 8 and Figure 9). To aid in validating results, the purine metabolism, regulation of autophagy, and regulation of actin cytoskeleton KEGG pathways were evaluated in ROC Plotter and were significantly able to discriminate cancer cells sensitive or resistant to pemetrexed based on transcriptome profiling (Figure S1) [25].

## 4. Discussion

The current study categorized cancer cell lines in the NCI 60-cell line panel as sensitive or resistant to antifolates based on available drug response data. By utilizing available baseline omics datasets from these cell lines in the NCI-60 cell line panel, molecular networks could be identified that differentiated sensitive and resistant cells, thereby uncovering molecular processes that are associated with antifolate response. Multiple approaches were used to define these processes, the first being a univariate approach which identified broad differences in RNA and ribosome processing. Deeper analyses revealed that a core network of molecules involved in metabolic processes, namely nucleotide metabolism and autophagy-related pathways, were strong differentiators of Q1 and Q4 cell lines. Differences in cell structure-related pathways, including cytoskeletal pathways, also coincided with these changes. Various measures were implemented to validate the findings in this study. Firstly, joint pathway analyses were conducted separately for metabolites + transcripts and metabolites + proteins, using both univariate and multivariate-selected molecules. Remarkably, this approach yielded consistent agreement between the two methods in identifying significant pathways across the omics datasets. The third validation method involved the integration of univariate and multivariate approaches along with pathway (metabolites + transcripts and metabolites + proteins) and network analysis approaches in the multi-omics analysis. These orthogonal approaches allowed for the identification of common key pathways and molecules, highlighting the importance of autophagy, cytoskeleton, and nucleotide metabolism-related pathways as key for differentiating cells sensitive or resistant to antifolates. Interestingly, the current study did not identify DHFR or TS as major differentiators of sensitive and resistant cells, but these have been primarily studied in acquired resistance settings. As our previous work has shown with alkylating agents, intrinsic and acquired resistance seem to be defined by distinct metabolic mechanisms, which may explain this difference [26,27,28].

The cytoskeleton and autophagy are two essential cellular processes that play critical roles in maintaining cell structure, function, and overall homeostasis. The cytoskeleton is a dynamic network of protein filaments found in the cytoplasm of eukaryotic cells, comprising microtubules, actin filaments (microfilaments), and intermediate filaments [29]. These filaments provide structural support, helping the cell to resist external forces and maintain its integrity. Moreover, the cytoskeleton is involved in intracellular transport, serving as tracks for motor proteins [30]. Motor proteins, such as dyneins and kinesins, move along the cytoskeletal filaments and transport various cellular components, including organelles, vesicles, and proteins, to their specific destinations within the cell [31]. This intracellular transport is essential for maintaining proper cellular organization and ensuring the correct distribution of cellular materials. Autophagy, on the other hand, is a highly regulated cellular process responsible for recycling and degrading unnecessary or dysfunctional cellular components. It plays a crucial role in maintaining cellular homeostasis by removing damaged organelles, misfolded proteins, and other cellular debris. Autophagy contributes to cellular quality control, ensuring that malfunctioning components are removed, and the cell remains healthy [32,33,34]. The process of autophagy involves the formation of double-membraned structures called autophagosomes. These autophagosomes engulf the cellular material targeted for degradation. Subsequently, the autophagosomes fuse with lysosomes, forming autolysosomes, where the enclosed material is broken down by lysosomal enzymes. The resulting breakdown products are then recycled back into the cytoplasm for reuse [35,36,37].

The connection between cytoskeleton activity and autophagy becomes evident when considering the process of autophagosome formation and intracellular transport. Autophagosome formation relies on certain components of the cytoskeleton, particularly microtubules, which provide the necessary tracks for the movement of motor proteins to transport vesicles and organelles throughout the cell [38]. The proper positioning and distribution of autophagosomes within the cell are crucial for efficient autophagy. Dynein motors move autophagosomes toward the cell center, close to the microtubule organizing center (MTOC), while kinesin motors move them toward the cell periphery. This bidirectional movement ensures that autophagosomes reach the appropriate regions of the cell for fusion with lysosomes and subsequent degradation [39]. Another connection between autophagy and the cytoskeleton is through mTOR signaling. The mTOR signaling pathway senses nutrient availability and can switch on autophagy during times of nutrient stress [40]. This pathway also integrates information about cellular nutrient and energy status with cytoskeletal dynamics, remodeling the actin cytoskeleton to facilitate invasion and metastasis [41,42]. Our data suggest that autophagy and the remodeling of cytoskeleton dynamics is playing a role in antifolate drug resistance.

The connection between autophagy and cancer drug resistance is a multifaceted and dynamic field of study within cancer biology. The role of autophagy in cancer is complex and context-dependent, as it can have both pro-survival and pro-death functions, depending on various factors including the stage of cancer, the specific tumor microenvironment, and the type of cancer treatment being administered [43]. Autophagy has emerged as a potential player in the development of drug resistance in cancer cells, and several mechanisms have been proposed to explain how autophagy may contribute to cancer drug resistance. Autophagy enables cancer cells to cope with cytotoxic stress by recycling cellular components and generating energy through the breakdown of intracellular contents. This enhanced cell survival allows cancer cells to withstand the toxic effects of the drugs and continue to proliferate, leading to drug resistance [44,45]. Cancer cells often experience high levels of cellular damage due to the aggressive nature of the disease and the exposure to therapeutic agents. Autophagy can play a protective role by selectively removing damaged organelles and proteins, preventing apoptotic signals which lead to cell death [46]. Autophagy can also influence cancer cell metabolism, allowing cells to adapt to nutrient-deprived conditions induced by anticancer drugs. By recycling cellular components, autophagy provides cancer cells with alternative energy sources and metabolic substrates, sustaining their survival and proliferation even under metabolic stress. This metabolic reprogramming may contribute to drug resistance by supporting cancer cell growth and resistance to therapeutic agents [47]. Despite the compelling evidence suggesting the involvement of autophagy in cancer drug resistance, the relationship is highly complex and context-specific. The effects of autophagy on cancer cell survival and drug response can vary based on factors such as the type of cancer, the stage of the disease, and the specific therapeutic agents used. Targeting autophagy, either to inhibit its pro-survival function or to enhance its pro-death function, has become an area of interest in the development of new anticancer treatments [48].

Aside from metabolic changes related to autophagy, the current study also identified purine metabolism as a major differentiator between sensitive and resistant cells. Cancer cells exhibit various mechanisms to rewire nucleotide metabolism and develop resistance to drug treatments [49]. By blocking DHFR, antifolates disrupt the production of nucleotide precursors, impairing DNA and RNA synthesis, ultimately leading to cell death [7]. As mentioned above, cancer cells can circumvent these effects and become resistant to antifolates through several adaptive mechanisms that involve rewiring enzymes in these pathways [8]. Our data suggest that cancer cells may activate alternative metabolic pathways for nucleotide synthesis, either through intracellular synthesis pathways or through the modulation of autophagy and its related pathways. Understanding these resistance mechanisms is crucial for developing strategies to combat drug resistance and improve the effectiveness of antifolate-based therapies. Targeting the altered components of nucleotide metabolism or employing combination therapies addressing multiple resistance mechanisms may provide more effective treatment options for overcoming drug resistance in cancer. Interestingly, this nucleotide metabolism alteration may be directly linked to the dysregulation of autophagy and the actin cytoskeleton. More research is needed to better understand how these three systems are linked and how this can be leveraged for new strategies to prevent or treat drug resistance to antifolates. This could potentially include inhibitors of purine metabolism given in combination with inhibitors of autophagy and cytoskeleton regulation, of which there are many [50]. Lastly, this methodology can be applied to other drug classes using these data sources, and it has recently been performed for the alkylating agents drug class [26].

## 5. Conclusions

In conclusion, this study employed a comprehensive approach to categorize cancer cell lines in the NCI 60-cell line panel as sensitive or resistant to antifolates based on drug response data and identified molecular networks that differentiate between these two groups. The analysis revealed significant associations between antifolate response and core molecular processes, particularly nucleotide metabolism and autophagy-related pathways, as well as changes in cell structure-related pathways, including cytoskeletal pathways. Understanding these resistance mechanisms is critical for developing effective strategies to combat drug resistance and enhance the efficacy of antifolate-based therapies. The findings of this study contribute valuable insights into the molecular underpinnings of antifolate response and open new avenues for exploring potential targets to overcome drug resistance in cancer treatment.

## Figures and Tables

**Table 1:** Median z-score rank orders of each cell line for antifolate agents in the NCI-60 cell line screen.

Cell Line	Cancer Type	Median z-Score Rank Order	Quartile Number
K-562	Leukemia	2	1
LOX IMVI	Melanoma	2.5	1
HCT-116	Colon	4	1
NCI-H460	Non-Small Cell Lung	4.5	1
786-0	Renal	5	1
CCRF-CEM	Leukemia	6	1
SW-620	Colon	8.5	1
HT29	Colon	9	1
HCT-15	Colon	9.5	1
ACHN	Renal	12	1
UACC-62	Melanoma	12.5	1
A549/ATCC	Non-Small Cell Lung	12.5	1
MCF7	Breast	13	1
OVCAR-8	Genitourinary	13	1
M14	Melanoma	14	1
MOLT-4	Leukemia	14.5	2
HL-60(TB)	Leukemia	15	2
SR	Leukemia	16	2
MDA-MB-435	Melanoma	17	2
SF-295	Central nervous system	18	2
SF-539	Central nervous system	20.5	2
CAKI-1	Renal	20.5	2
SF-268	Central nervous system	21.5	2
SN12C	Renal	21.5	2
HCC-2998	Colon	22	2
DU-145	Genitourinary	22	2
NCI/ADR-RES	Genitourinary	23	2
SK-MEL-5	Melanoma	24	2
KM12	Colon	25.5	2
HOP-62	Non-Small Cell Lung	25.5	2
NCI-H23	Non-Small Cell Lung	26	3
U251	Central nervous system	28.5	3
IGROV1	Genitourinary	29	3
PC-3	Genitourinary	29	3
RPMI-8226	Leukemia	31	3
UO-31	Renal	32	3
NCI-H522	Non-Small Cell Lung	33	3
SNB-19	Central nervous system	36.5	3
NCI-H322M	Non-Small Cell Lung	36.5	3
OVCAR-5	Genitourinary	36.5	3
UACC-257	Melanoma	37	3
A498	Renal	38	3
COLO 205	Colon	39.5	3
OVCAR-3	Genitourinary	39.5	3
SK-MEL-28	Melanoma	42	4
MALME-3M	Melanoma	42.5	4
TK-10	Renal	43	4
EKVX	Non-Small Cell Lung	45.5	4
SK-OV-3	Genitourinary	45.5	4
T-47D	Breast	47	4
HOP-92	Non-Small Cell Lung	47	4
RXF-393	Renal	47	4
BT-549	Breast	48	4
SNB-75	Central nervous system	48	4
NCI-H226	Non-Small Cell Lung	49	4
OVCAR-4	Genitourinary	49.5	4
MDA-MB-231/ATCC	Breast	53	4
HS 578T	Breast	54	4

Missing z-scores indicate that the corresponding cell line did not have z-score information for a given compound. A lower rank order indicates a higher z-score (greater sensitivity to a compound). Compounds that did not have experimental data after quality control were removed. NSC = NCI’s accession number for test compounds. Ties were not broken for cell lines that had the same z-score for a given NSC.

**Table 2:** Top 15 pathways by *p*-value identified from multi-omic network analysis comparing Q1 and Q4.

Pathway	*p*-Value	FDR
EGFR tyrosine kinase inhibitor resistance	1.03 × 10^−165^	3.45 × 10^−163^
Glycolysis/Gluconeogenesis	3.16 × 10^−28^	5.30 × 10^−26^
Endocrine resistance	1.84 × 10^−24^	2.06 × 10^−22^
ABC transporters	1.17 × 10^−23^	9.82 × 10^−22^
Arginine and proline metabolism	6.36 × 10^−17^	4.27 × 10^−15^
Pyrimidine metabolism	3.42 × 10^−16^	1.92 × 10^−14^
beta-Alanine metabolism	8.10 × 10^−16^	3.89 × 10^−14^
Platinum drug resistance	4.10 × 10^−14^	1.72 × 10^−12^
Purine metabolism	6.76 × 10^−14^	2.52 × 10^−12^
Lysine degradation	2.39 × 10^−12^	8.04 × 10^−11^
Amino sugar and nucleotide sugar metabolism	4.93 × 10^−12^	1.50 × 10^−10^
Glutathione metabolism	2.07 × 10^−11^	5.79 × 10^−10^
Pentose phosphate pathway	4.08 × 10^−11^	1.06 × 10^−9^
Histidine metabolism	6.18 × 10^−11^	1.48 × 10^−9^
Fatty acid degradation	6.89 × 10^−11^	1.54 × 10^−9^

**Table 3:** DIABLO-selected variables that had an AUC > 90% in ROC curves predicting Q4 status.

Gene Symbol	AUC	Log2 FC
NOB1_T	0.986	1.13
RSL24D1_T	0.967	0.89
TRAP1_T	0.957	0.93
MYOC_T	0.948	0.16
SNX11_Me	0.940	−0.14
LOC101930306_T	0.924	0.33
STK26_T	0.924	1.95
ZNF500_T	0.924	0.27
DHODH_T	0.919	0.75
234900_at_T	0.914	0.20
C10orf2_T	0.914	0.82
IGFBP7_T	0.914	−2.46
DDX51_T	0.910	0.65
E2F4_T	0.910	0.50
NAA25_T	0.910	0.63
NIP7_T	0.910	0.77
NPM3_T	0.910	0.69
PHB2_T	0.910	0.72
SNX21_T	0.910	−0.64
UBE2I_T	0.910	0.34
CAPN15_T	0.905	0.64
HNRNPA1_T	0.905	0.85
SNHG4_T	0.905	0.70
SNORA52_T	0.905	0.39
SRSF12_Me	0.905	1.50

Variables ending in “_T” indicate those that are derived from the transcriptomics dataset and variables ending in “_Me” indicate those that are derived from the DNA methylation dataset.

**Table 4:** Top 10 pathways from joint pathway analysis of metabolites and transcripts selected by DIABLO.

Metabolic Pathways Only	
Pathway	*p*-Value
Purine metabolism	0.009219
Glycerophospholipid metabolism	0.035887
Sphingolipid metabolism	0.052286
Arginine biosynthesis	0.058987
One carbon pool by folate	0.075348
Glycerolipid metabolism	0.092975
Glycosaminoglycan biosynthesis—heparan sulfate/heparin	0.098934
Glycosaminoglycan degradation	0.136300
Nitrogen metabolism	0.138360
Phenylalanine, tyrosine and tryptophan biosynthesis	0.151120
**All Pathways**	
Pathway	*p*-value
Lysosome	7.29 × 10^−7^
Phagosome	8.48 × 10^−7^
Focal adhesion	8.02 × 10^−5^
Vibrio cholerae infection	0.000319
Regulation of actin cytoskeleton	0.000673
Ribosome	0.000704
Autophagy—animal	0.00191
ECM-receptor interaction	0.00279
Mitophagy—animal	0.003552
Human papillomavirus infection	0.006515

**Table 5:** Top 10 pathways from joint pathway analysis of metabolites and proteins selected by DIABLO.

Metabolic Pathways Only	
Pathway	*p*-Value
Purine metabolism	0.019147
Arginine biosynthesis	0.095444
Pentose and glucuronate interconversions	0.112200
Citrate cycle (TCA cycle)	0.144890
Pyruvate metabolism	0.154490
Pentose phosphate pathway	0.160830
Glycolysis or Gluconeogenesis	0.204070
Cysteine and methionine metabolism	0.233730
Amino sugar and nucleotide sugar metabolism	0.256740
Steroid biosynthesis	0.265210
**All Pathways**	
Pathway	*p*-value
ABC transporters	5.65 × 10^−6^
Regulation of actin cytoskeleton	1.54 × 10^−5^
Tight junction	6.31 × 10^−5^
Leukocyte transendothelial migration	0.000202
Platelet activation	0.000393
Focal adhesion	0.001603
Pathogenic Escherichia coli infection	0.001693
Ribosome biogenesis in eukaryotes	0.002361
Phagosome	0.006792
Oxytocin signaling pathway	0.008358

## Data Availability

All data can be found in the Supplementary Materials and the provided links to public datasets.

## Supplementary Materials

The following supporting information can be downloaded at: https://www.mdpi.com/article/10.3390/biomedicines11092532/s1, Table S1: List of z-score rank orders for antifolate compounds in the NCI-60 cell line screen; Table S2: *p*-values and fold changes of all variables between Q1 and Q4. Table S3: Full pathway results from joint pathway and network analyses using metabolites, transcripts, and proteins with *p* < 0.1 between Q1 and Q4. Table S4: Loadings values for the first 5 principal components of the DIABLO model for all variables. Table S5: Full joint pathway analysis results using metabolites and transcripts or metabolites and proteins selected by the DIABLO model. Figure S1: ROC Plotter results of pathways that predict response to pemetrexed.
