# Supplementary material for: Unlocking the Molecular Secrets of Antifolate Drug Resistance: A Multi-Omics Investigation of the NCI-60 Cell Line Panel
Source: Biomedicines. 2023 Sep 14;11(9):2532. doi: 10.3390/biomedicines11092532 (PMC10526174; doi:10.3390/biomedicines11092532)
Supplement: Supplementary file 1 [file biomedicines-11-02532-s001.zip › Supplemental Material/Supplemental Figure S1.pptx]

## Slide 1
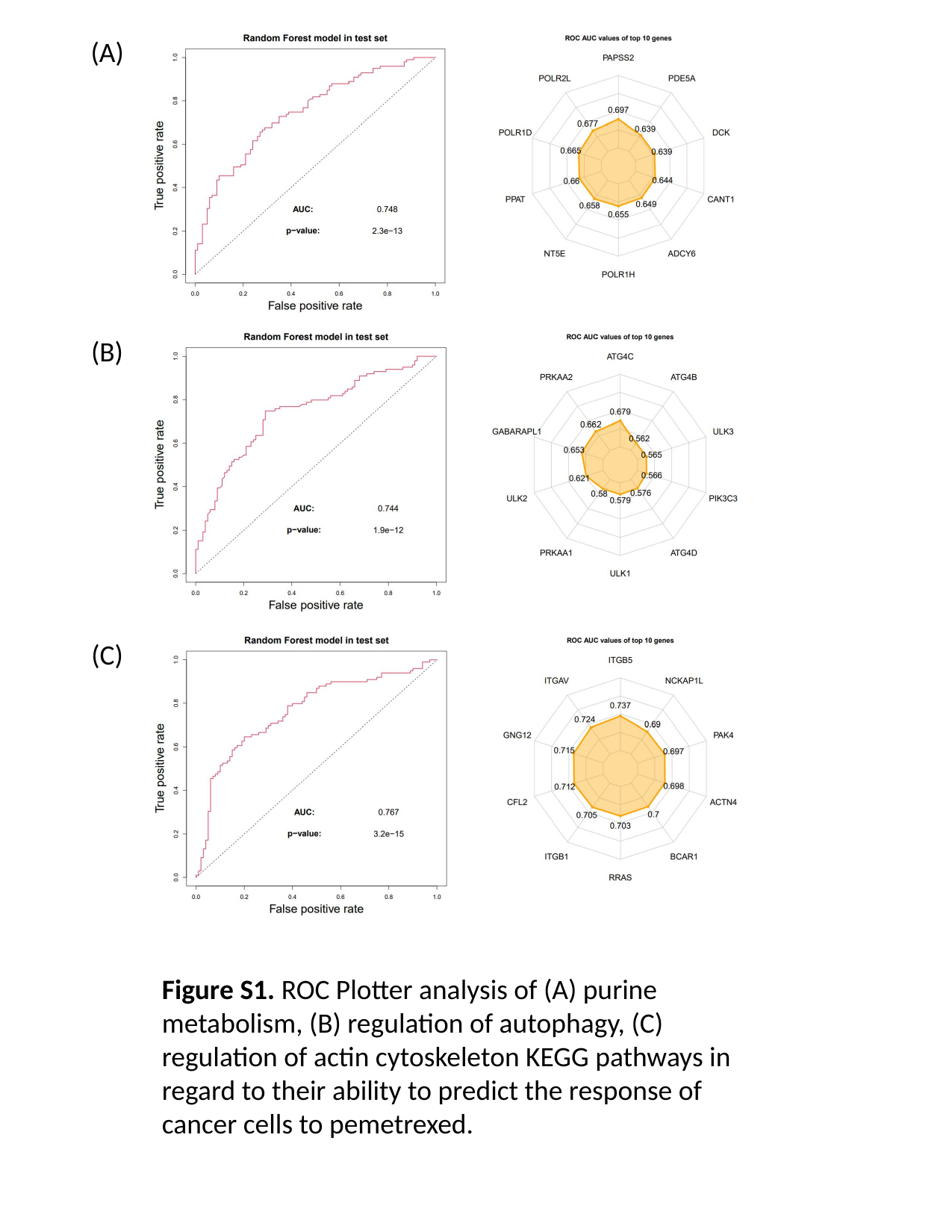

(A)
(B)
(C)
Figure S1. ROC Plotter analysis of (A) purine metabolism, (B) regulation of autophagy, (C) regulation of actin cytoskeleton KEGG pathways in regard to their ability to predict the response of cancer cells to pemetrexed.
